# Investment case for malaria elimination in South Africa: a financing model for resource mobilization to accelerate regional malaria elimination

**DOI:** 10.1186/s12936-021-03875-z

**Published:** 2021-08-16

**Authors:** Joseph Njau, Sheetal P. Silal, Aparna Kollipara, Katie Fox, Ryleen Balawanth, Anthony Yuen, Lisa J. White, Mandisi Moya, Yogan Pillay, Devanand Moonasar

**Affiliations:** 1 JoDon Consulting Group, 4501 Forest View Court, Lilburn, GA 30047 USA; 2 Modelling and Simulation Hub, Africa (MASHA), Department of Statistical Sciences, University of Cape Town, Cape Town, South Africa; 3 Nuffield Department of Medicine, Centre for Global Health, Oxford University, Oxford, UK; 4 Health Economist and Health Financing Specialist, California Public Health Department, Sacramento, USA; 5 Department of Global Health at the School of Medicine and Packard Foundation, University of California San Francisco, San Francisco, CA USA; 6 Clinton Health Access Initiative (CHAI), South Africa Regional Office, Pretoria, South Africa; 7 Big Data Institute, Nuffield Department of Medicine, Li Ka Shing Centre for Health Information and Discovery, University of Oxford, Oxford, UK; 8 Center for Innovation in Global Health, Georgetown University, Georgetown, USA; 9 Malaria Vector and Zoonotic Disease Directorate, National Department of Health, Pretoria, South Africa; 10 School of Health Systems and Public Health, University of Pretoria, Pretoria, South Africa

## Abstract

**Background:**

Malaria continues to be a public health problem in South Africa. While the disease is mainly confined to three of the nine provinces, most local transmissions occur because of importation of cases from neighbouring countries. The government of South Africa has reiterated its commitment to eliminate malaria within its borders. To support the achievement of this goal, this study presents a cost–benefit analysis of malaria elimination in South Africa through simulating different scenarios aimed at achieving malaria elimination within a 10-year period.

**Methods:**

A dynamic mathematical transmission model was developed to estimate the costs and benefits of malaria elimination in South Africa between 2018 and 2030. The model simulated a range of malaria interventions and estimated their impact on the transmission of *Plasmodium falciparum* malaria between 2018 and 2030 in the three endemic provinces of Limpopo, Mpumalanga and KwaZulu-Natal. Local financial, economic, and epidemiological data were used to calibrate the transmission model.

**Results:**

Based on the three primary simulated scenarios: Business as Usual, Accelerate and Source Reduction, the total economic burden was estimated as follows: for the Business as Usual scenario, the total economic burden of malaria in South Africa was R 3.69 billion (USD 223.3 million) over an 11-year period (2018–2029). The economic burden of malaria was estimated at R4.88 billion (USD 295.5 million) and R6.34 billion (~ USD 384 million) for the Accelerate and Source Reduction scenarios, respectively. Costs and benefits are presented in midyear 2020 values. Malaria elimination was predicted to occur in all three provinces if the Source Reduction strategy was adopted to help reduce malaria rates in southern Mozambique. This could be achieved by limiting annual local incidence in South Africa to less than 1 indigenous case with a prediction of this goal being achieved by the year 2026.

**Conclusions:**

Malaria elimination in South Africa is feasible and economically worthwhile with a guaranteed positive return on investment (ROI). Findings of this study show that through securing funding for the proposed malaria interventions in the endemic areas of South Africa and neighbouring Mozambique, national elimination could be within reach in an 8-year period.

**Supplementary Information:**

The online version contains supplementary material available at 10.1186/s12936-021-03875-z.

## Background

**Fig. 1:**
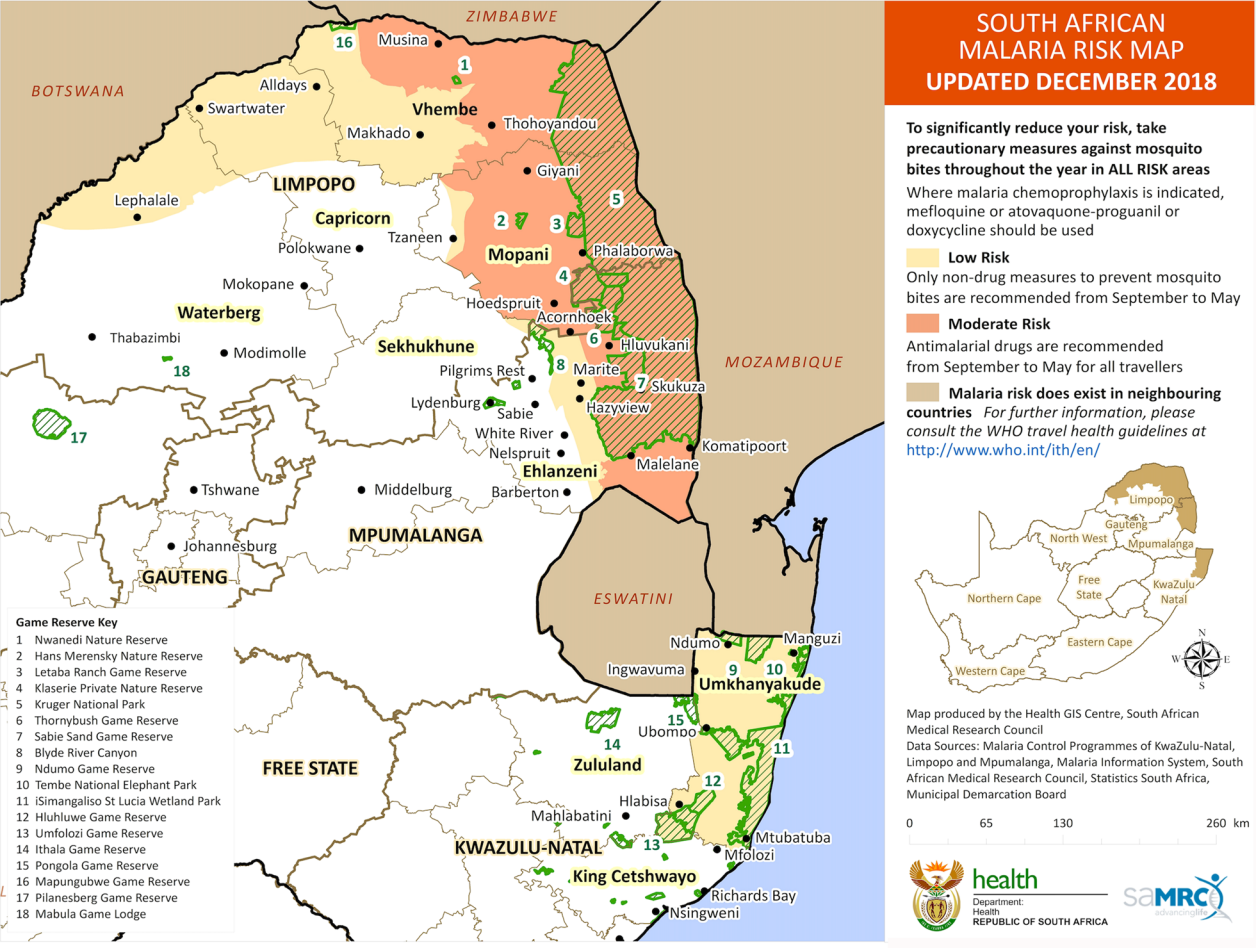
South African malaria risk map 2018 [4]

Despite malaria being confined to only three provinces –Limpopo, Mpumalanga and KwaZulu-Natal (KZN) in South Africa, the disease continues to pose serious public health threats nationwide (Fig. 1). Since 2010, South Africa has made significant strides in combatting the burden of malaria from within its borders. However, during the last two decades, South Africa experienced at least two major malaria outbreaks that resulted in hundreds of hospital admissions and deaths. The first outbreak which was mainly concentrated in KwaZulu-Natal province occurred in early 2000 where over 60,000 cases were recorded [1]. The most recent malaria outbreak was reported in 2017, when over 28,000 cases were recorded in South Africa with Limpopo province accounting for majority of new malaria transmissions [2, 3]. These sporadic malaria outbreaks continue to be a large public health concern. Cross-border migration from neighbouring countries is a major contributor to malaria transmission in South Africa where importation of cases has fueled local malaria transmission. For instance, in the last five years, most malaria cases in Mpumalanga and KZN provinces were imported from neighbouring countries. Malaria importation accounted for 82% and 72% of total cases in Mpumalanga and KZN, respectively [4].

Following sporadic malaria outbreaks that started in early 2014 to 2017, six southern African countries experienced a dramatic increase in cases [5]. Cross-border migration of mobile and migrant populations (MMPs) in the region poses a significant challenge for malaria elimination efforts in the region, indicative of a need for a stronger regional response to this disease. In keeping with WHO’s Global Malaria Elimination agenda, all 16 SADC Heads of State signed the Windhoek Declaration on Eliminating Malaria in the SADC Region on 18 August 2018, committing to the collective goal of malaria elimination in the region [6, 7]. The Declaration also commits to the intensification of cross-border collaborations, measures to improve data-sharing and programme implementation, and increased resource allocation from country governments to address funding gaps.

Population movement from neighbouring countries, particularly Mozambique, is of relevance. In KZN alone, 70% of all malaria cases recorded in 2017 were imported from Mozambique. Given the substantial role of cross-border migration in South Africa, achieving national malaria elimination can enhance the country’s regional malaria efforts and improve global health security. In recent years, global health security has emerged as a prominent global health agenda item, as it acts to protect the collective health of populations across borders. Health security is thus experienced as a regional good, requiring regional cooperation. More recently, especially in the context of COVID-19 the concepts of global health solidarity and sustainability have also seen increased urgency.

The risk of malaria spreading across borders is particularly high for low and middle-income countries, whose health systems are generally less equipped to prevent and respond to public health threats and because of economic reasons as people move across borders in search for economic opportunities. It is within this context that an investment case for malaria elimination in South Africa was conceived. The investment case argues for source reduction in Southern Mozambique as a necessary component in the strategy to achieve malaria elimination in South Africa and ultimately in the countries forming the Southern African Regional Economic Development Cooperation or (SADC).

While supporting malaria elimination commitments, funding for malaria control in South Africa continues to be limited as the country is ineligible to receive any funding for malaria from Global Fund for HIV/AIDS, TB and Malaria (GFATM). Only regional malaria programmes are funded as South Africa is classified by the World Bank as an upper middle-income country. [8]. According to South Africa’s Department of Health, the country is only eligible to receive external funding through two multi-country GFATM grants: MOSASWA (Mozambique, South Africa and Eswatini) and the Elimination 8 Initiative (E8) (established in 2015). Through this external support, the National Malaria Programme (NMP) in South Africa has been able to establish 15 border units to promptly detect and treat cases among local and mobile and migrant populations with most of these clinics being established from 2017 onwards. The Global Fund support for the border units in South Africa ended in December 2019 (E8) while funding for the other units from the MOSASWA grant is expected to end in December 2022 [6, 9]. As part of the investment case and resource mobilization strategy, the government of South Africa made the decision to integrate the border units into the provincial malaria programmes through domestic financing. These border units are currently filling a critical gap for case management and surveillance activities in strategic, high transmission areas of Limpopo and Mpumalanga and residual focal transmission areas in KZN. The increasing challenge of imported malaria cases in the three endemic provinces requires innovative, proactive surveillance measures to promptly detect and treat imported cases, effective treatment to prevent severe illness and deaths, and sustained health promotion to increase awareness of malaria prevention strategies. Reducing malaria importation to the foci of transmission within endemic provinces with an effective intervention such as the border units will strengthen the country’s elimination efforts.

It is in this context that a malaria investment case study was conceived by the government of South Africa through its National Malaria Programme and in collaboration with its international partners. The goal of the study was to conduct a cost–benefit analysis and the financial feasibility of malaria elimination in South Africa. The investment case aimed to inform malaria programme budgeting and strategic planning, mobilize resources domestically and internationally through increased financial and political commitment, and catalyze advocacy in South Africa, and the region, for malaria elimination. Additionally, the study aimed to make a convincing data-driven case to decision makers in government and the development community to increase investments towards malaria elimination while maximizing economic returns in the long-run.

## Methods

A mathematical transmission model was developed to estimate the costs and benefits of malaria elimination in South Africa between 2018 and 2030. The model simulated a range of malaria interventions and estimates their impact on the transmission of *Plasmodium falciparum* malaria between 2018 and 2030 in the three endemic provinces. Expert opinion from the South African Investment Case Technical Task Team, nominated by the NMP Director, was used to select interventions and scenarios to model (chief selection criteria included availability of effectiveness data per intervention and the feasibility of implementing new interventions). The economic analysis was informed by the outputs and the malaria transmission model developed by the University of Cape Town’s Modelling and Simulation Hub, Africa (MASHA). The model was adapted from previously published studies on the prospects of malaria control and elimination in South Africa [10, 11] and other countries [12, 13]. The costs and benefits associated with achieving elimination were inflated to mid-year 2020 South African Rand values and consequently converted to 2020 midyear US dollars market exchange rate.

### Study site

The transmission model was populated with demographic and epidemiological data from districts in the three malaria endemic provinces of Mpumalanga, Limpopo and KZN. In Limpopo, Vhembe and Mopani Districts experience the highest malaria transmission. Waterberg, Sekhukhune and Capricorn districts have low local transmission, but are vulnerable to increased transmission, as demonstrated during the 2017 outbreak. Ehlanzeni is the only endemic district in Mpumalanga, of which Bushbuckridge and Nkomazi municipalities experience the highest transmission. In KZN, malaria transmission primarily occurs in Umkhanyakude District with very low transmission in Zululand and King Cetshwayo Districts. Both Mpumalanga and KZN border Mozambique, resulting in high importation of malaria and subsequent local transmissions [4]

### Data sources

Financial, economic, and epidemiological data were collected to populate the transmission model. A literature review was conducted to obtain an in-depth understanding of the current malaria situation in South Africa, as well as the financial landscape for malaria. Historical malaria budget and expenditure data were obtained from the South African National Treasury and the NMP. Key stakeholder interviews were conducted with Department of Health officials involved in malaria activities at the national and provincial levels, relevant South Africa-based partners from academia and NGOs, and other key government stakeholders involved in resource mobilization for health. Cost data were obtained from a variety of sources in each of the three provinces, including provincial malaria programme operational plans, the national IRS micro plan, the National Department of Health master procurement catalogue for medicines, the price list from the National Health Laboratory Service and relevant National Department of Health tender documents. The unit cost data on the E8/MOSASWA-funded border units were calculated based on budgets from the implementing partner, Humana People to People (HPP). The type and quality of data varied across interventions and fall into three categories: actual expenditures from preceding years; prospective budgets or funding requests based on previous requests, population, or expert opinions; or recent experience of implementing the intervention.

### Calculation of the economic burden of malaria

The total economic burden of malaria in the three malaria-endemic provinces of South Africa was assessed and estimated in 2018 and in this report, the estimates were inflated to mid-year 2020 prices. This was also calculated as the share of South African annual gross domestic product (GDP). To estimate the total economic burden of malaria, the following components were evaluated: (1) direct cost to the health system; (2) direct cost to the household; and (3) indirect cost to society which mainly focused on the opportunity costs of being treated for malaria or, caring for malaria patients as outlined in Table 1. The indirect costs (statistical value of life) resulting from premature death was calculated based on the methods developed by the Institute for Health Metrics and Evaluation (IHME) group [14]. The methodology has been validated through various World Health Organization (WHO) programmes, including the Roll Back Malaria (RBM)’s -Action and Investment to defeat Malaria (AIM) report [15]. Details on the cost components included in this investment case are provided in Additional file 1.

**Table 1:** Malaria cost components

Direct cost to the health system	Direct cost to individual households	Indirect cost to society
1. Cost of malaria diagnosis, testing and treatment for both outpatients and inpatients 2. Cost of vector control and other malaria control sensitization campaigns 3. Cost of intermittent malaria prevention in pregnant women & seasonal malaria chemotherapy in children 4. Cost of conducting malaria surveillance 5. Cost of malaria programme planning activities	1. Out-of-pocket (OOP) expenditure incurred due to malaria a. Hospital-based costs such as consultation, laboratory tests, drugs, and admission costs b. Non-hospital costs such as transport, food, and lodging c. OOP expenditure for malaria prevention activities	1. Cost due to loss of life due to malaria mortality 2. Cost due to loss of productivity due to malaria morbidity 3. Loss of income due to taking care of malaria patients

Given that the South African public health sector does not maintain facility level cost data per type of admission, inpatient costs of malaria case management were extrapolated from average cost per patient day equivalent data for each level of care. Medicines and laboratory expenditure was removed from the cost per patient day equivalent (PDE) calculation and populated with malaria specific costs for medicines and laboratories. The total cost per inpatient admission was determined based on clinical expert advice on the average length of stay for an inpatient malaria case. Assumptions were based on the cost per PDE in public hospitals (incorporating malaria treatment and screening costs). Expert advice from malaria specialist physicians and the South African Malaria Treatment Guidelines were used to determine appropriate malaria treatment regimens, staff time used to attend malaria patients, and their associated costs. Meanwhile, out of pocket (OOP) costs were calculated based on estimated transport costs to seek care at an outpatient and inpatient facility and the hospital fees related to an inpatient admission. The household costs of malaria treatment (OOP expenditures) were estimated based on a study conducted in KZN in early 2000 [16]. These costs were extrapolated to 2017 prices, which may not necessarily represent the actual costs that households face today, given changes to the malaria treatment regimen and travel costs to and from facilities.

**Table 2:** List of interventions included in the mathematical model

Intervention	Description
Passive Case Detection	Uncomplicated malaria treated with Artemether lumefantrine through public health facilityes and the private sector based on treatment seeking data and KAP studies
	Severe malaria treated in hospital with IV Quinine and IV artesunate determined by data
Indoor Residual Spraying	Modelled by insecticide type (DDT, non-DDT) as coverage determined by the number of structures sprayed (data informed) and the population at risk in need of spraying. In consultation with the technical task team entomologists and vector surveillance teams, the waning in effectiveness of the sprays was modelled in line with the WHOPES recommendations. The main vector responsible for malaria transmission in South Africa (*Anopheles arabiensis*) is known for its outdoor biting and resting behavior. By accounting for probabilities of indoor biting, indoor resting, repelling and killing effectiveness, effectiveness (for a newly sprayed structure) is computed at 38%. This effectiveness will decrease with the cumulative IRS coverage over time. These numbers have been sourced from existing literature and verified/adjusted by the Technical task team The success of IRS with DDT is well documented in South Africa with a decrease in incidence of 91% experienced following the reintroduction of DDT (combined with introduction of ACTs) after the 2000 epidemic {Hargreaves, 2000 #23; Maharaj, 2005 #24; Sharp, 2007 #22} DDT has been successfully used in South Africa for decades and was the primary reason for the significant decline in cases during the 1996–2000 epidemic. DDT was used to reduce the population of Pyrethroid-resistant *Anopheles funestus* vectors and is still being used to drive back the spread of these mosquitoes that are prevalent in neighboring countries
Active Case Detection	Modelled for districts within Mpumalanga and KwaZulu-Natal. Not active in Limpopo currently, but included as incidence is projected to decrease by 2022 due to all risk spraying activities under the Accelerate and Source Reduction scenarios
Proactive Case Detection	Modelled for districts within Mpumalanga and KwaZuluNatal. Not active in Limpopo currently as incidence is too high. Malaria Surveillance Agents (MSAs) take 3 slides per day and follow up positive cases to receive treatment in KwaZulu-Natal. Also includes screening and testing in areas which may have high numbers of asymptomatic carriers such as seasonal farming areas and mining communities
Border Surveillance	Border units are operational in certain districts within Limpopo, Mpumalanga and KwaZulu-Natal (South African side only) and their detection of positive cases is modelled in line with the data received from the physical units

### Estimating overall programme costs

Finally, different malaria elimination programme activities were used to estimate overall total programme costs. Such activities included IRS, active/reactive case detection, case investigation, surveillance, training and Information, Education, and Communication (IEC) materials, programme administrative expenses, facility-based case management costs as well as household out of pocket expenses. These interventions were selected based on their expected efficacy in malaria control and elimination as recommended by Technical Task Team. Malaria control activities associated with implementation of these interventions were aggregated to generate estimates on overall costs of malaria elimination over the 11-year period.

### Epidemiological-economic transmission model

A bespoke epidemiological-economic transmission model was developed to simulate malaria transmission in South Africa to predict the impact and determine the cost of current and proposed interventions against *P. falciparum* malaria. Given that previously set elimination targets of 2018 and 2020 were unlikely to be met, the model set out to assess if elimination could be achieved through different combinations of interventions. This model, adapted from application in other countries to the South African local context, is an addition to the Malaria Elimination Transmission and Costing (METC)-Country Suite, called METC-South Africa (METC-SA) [12, 13].

The METC-Country suite is a set of mathematical models developed by the Oxford Modelling for Global Health (OMGH, Oxford University) and the Modelling and Simulation Hub, Africa (MASHA, University of Cape Town) to guide national and regional malaria control and elimination efforts. These models combine epidemiological and cost data collection, curation and analysis with multi-species and single-species, spatially explicit transmission models of differing complexity. As such, they may be used to predict both the health outcomes and costs associated with various programme options for achieving a given malaria elimination strategy. The characteristics and additional details of the model are provided in Additional file 1.

### Defining population at risk (PaR)

Population data was available down to the local municipality level. The population at risk (PaR) of local malaria transmission and those eligible for IRS in each of the districts was computed based on areas of sustained local transmission and is routinely assessed by the NMP and provincial programmes. However, during the 2017 outbreak, local cases were observed to occur in low-risk local municipalities that were thought to contain cleared foci. Therefore, the PaR has been computed in two forms for use in the model: (i) IRS PaR: Population living in local municipalities that exhibited sustained local endemicity by reporting at least one local case every year from 2015 to 2017 and (ii) Transmission PaR: Population living in local municipalities exhibiting risk of local infection by reporting at least one local case in the 2017 outbreak (the largest outbreak observed since 2000).

The Transmission PaR will be larger than the IRS PaR as it comprises a population that also benefits indirectly from IRS implemented in higher risk neighbouring areas. The IRS PaR is conservative in the sense that it considers only populations that maintain consistent local transmission thereby being candidates for IRS intervention, whereas the Transmission PaR considers populations where it is possible for sporadic local transmission to occur in years of high transmission (e.g. 2017). PaR is assumed to remain constant over the simulation period.

### Classification of cases

Given that the connectivity between endemic areas determines the transmission level in each area, a requirement for the model is that all cases should be classified by source of infection. To correctly model the true magnitude of reported cases, it was assumed that the unclassified cases (due to incomplete records, untraceable patients during the case investigation) were unbiased in their source, i.e., unclassified cases were attributed proportionally to the distribution of classified cases. Scenarios where a scale-up of interventions is modelled include improved case classification activities in Limpopo and KZN (Mpumalanga has not reported many unclassified cases in the last three years due to classifications from both case notification and case investigation being used).

### Uncomplicated vs. severe cases

Severe cases requiring hospitalization were determined by inpatient status and the drug used for treatment reported in the Malaria Information System (MIS), in Mpumalanga and KZN. In the absence of MIS data, severe cases were defined by treatment acquired per facility type (Limpopo). While some inpatient cases may likely have been uncomplicated, nevertheless, in the absence of additional information and for costing purposes, an assumption was made that 50% of all cases seen at hospitals in Limpopo constituted uncomplicated malaria cases. This is corroborated by reports of stockouts during outbreaks in outpatient facilities resulting in the treatment of uncomplicated cases in hospital.

### Interventions to be modelled

Modelling was guided by the current guidance on malaria case management. Interventions considered in the model are described in Table 2.  For instance, the consideration to shift from intravenous quinine to intravenous artesunate as gold standard treatment for severe malaria case management in high burden areas was considered in limited settings. Intravenous artesunate is the current recommended treatment of severe malaria. Several hospitals in the endemic provinces had commenced implementing the treatment, but it was yet to be adopted in all hospitals. Through lowering mortality in patients with severe malaria and decreasing the duration of infectiousness, several analyses and a meta-analysis of trials comparing IV artesunate and IV quinine strongly suggest that parenteral artesunate should replace quinine as the treatment of choice for severe falciparum malaria worldwide [17]. Nevertheless, since this recommendation is currently being gradually adopted it was modelled with limited effectiveness.

Secondly, a recent review of Single Low Dose (SLD) primaquine studies concluded that a single low dose of primaquine added to an artemisinin regimen for malaria reduces infectiousness to mosquitoes though it was unclear whether this reduction would materially reduce malaria transmission in communities [18]. The review suggested that SLD Primaquine reduces infectiousness in mosquitoes by 88% on day 3 or 4, but with a wide confidence interval (0.12, 0.98) due to the inconclusiveness of studies. Although the effectiveness of primaquine as an addition to ACT is widely agreed upon, in the current model, it was not possible to estimate its impact on transmission. This recommendation was, therefore, not included in the modelled scenarios.

In addition to these model limitations, the following interventions were not considered in the scenarios to be modelled:

i. Mass community drug administration (MDA) and screening were not considered because the current malaria policy in South Africa does not include this in the national strategy. Such strategies can, therefore, only be implemented with a change in the national malaria policy. However, should South Africa decide to pursue this route, the model can be adapted to accommodate such changes.
ii. Larviciding activities: Following the advice of investment case technical task team, it was decided that though the impact of larviciding was yet to be evaluated, the cost of larviciding be included in the model. The benefits may be underestimated because those associated with larviciding are not accounted for, even though the costs of implementing larvicide are incorporated in the model.
iii. At the time of developing the model, the use of prophylaxis among high-risk populations was also not included as it was not part of the 2012–2018 malaria policy guidelines. This has subsequently been included in the 2019–2023 National Strategic Plan (NSP) for malaria elimination. While the inclusion of targeted prophylaxis may increase costs, it could have significant benefits by reducing onward transmission of malaria and morbidity rates in high risk populations.
iv. Finally, the distribution of long-lasting Insecticide-treated Nets (LLINs) was not considered in the South Africa’s malaria investment case model for the following reasons: The behaviour of the major vector responsible for current malaria transmission in South Africa is *Anopheles arabiensis*. This mosquito is an opportunistic biter exhibiting both exophagic (outdoor biting) and exophilic (outdoor resting) behaviour with a 60% outdoor biting rate [19]. The consistently high coverage of IRS is already affording effective protection indoors and thus, LLINs would only provide additional (if not complementary) protection indoors [20, 21]. Secondly*,* South Africa’s intent is to rely on IRS as a primary vector control method for community level protection. For the strategy to succeed it requires the highest possible coverage to achieve the maximum protection against malaria transmission. The WHO already recommends the use of IRS only in those settings where it can be implemented effectively, relying on a high and sustained level of political commitment, which South Africa has demonstrated in the last 20 years. LLINs may be considered as a form of protection for migrant/mobile populations. These populations are difficult to trace, and it is not well known where the populations linger and how they move about upon testing positive with malaria at a detection site. Due to uncertainties around its implementation and effectiveness, South Africa’s malaria investment case technical team recommended that NMCP provide prophylaxis with Single Low Dose Primaquine to migrant populations for the prevention of onward transmission. The impact of replacing IRS with LLINs would need to be assessed through modelling at a micro-population level where differences in strip-spraying, barrier-spraying and others would be evaluated and compared to LLIN usage in small communities that experience different migration rates. Since such evaluation was beyond the scope of this investment case, it was determined that such evaluations can be considered in future micro-level modelling exercise.

### Other scenarios modelled with limited effectiveness

In consultation with the NMP and the Technical Task team, the modelling team considered a variety of interventions (recommended by the WHO) to be included in the scenarios presented in Table 3. The current set of interventions and the levels at which they are being implemented were determined based on a review of the National Strategic Plan: 2012–2018 Malaria Elimination Strategy for South Africa and through engagements with the NMP and provincial malaria programmes. The *Business as usual* scenario assumed that the existing set of activities (which in this case excludes targeted prophylaxis) will continue at current levels in each of the three provinces with endemic malaria transmission.

**Table 3:**
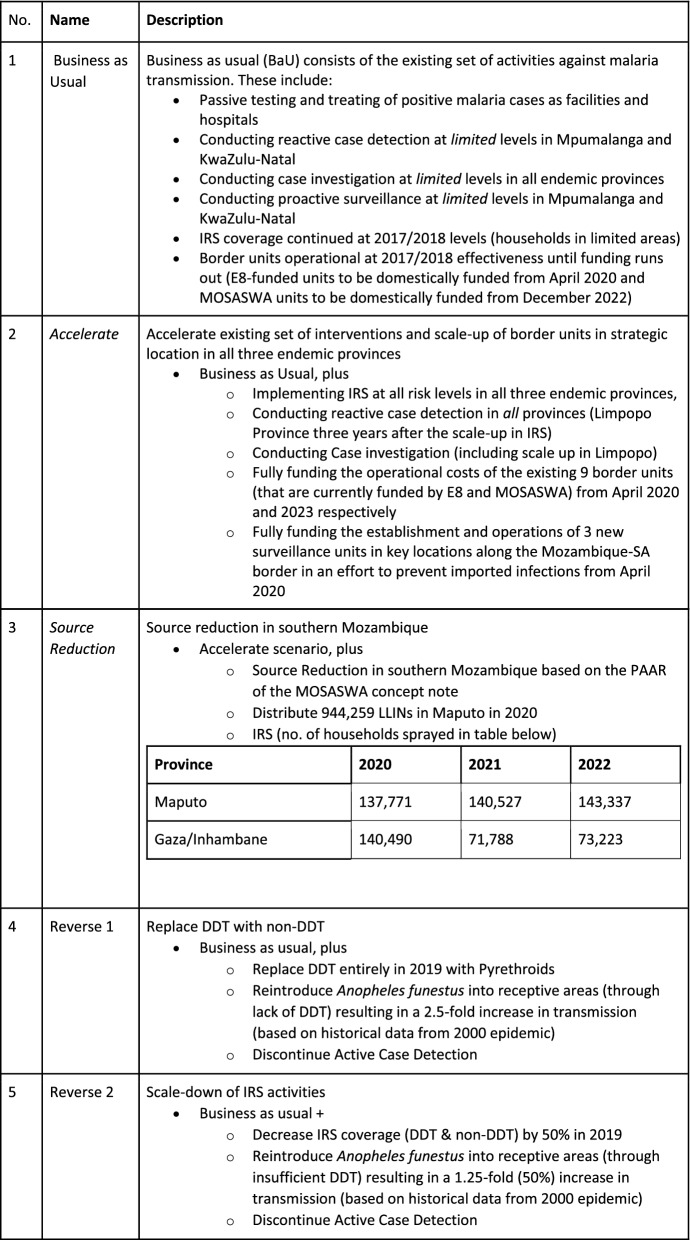
Modelled scenarios with descriptions

The proposed *Acceleration scenario* was based on a study conducted by the NMP to determine the implementation and cost gap in the IRS programme and case management activities in the endemic provinces. The scenario reflects a scale-up of current activities at levels agreed upon by the NMP and the provincial malaria programmes. Additionally, given the high level of imported cases in Mpumalanga and KZN, it was decided to explore the option of establishing more border units in these provinces.

The *Source reduction scenario* reflects the scale-up of interventions considered in the *Accelerate scenario* and simulates an aggressive approach to reducing imported cases by implementing source reduction activities in southern Mozambique. As part of discussions for the MOSASWA Global Fund application, a list of Prioritized Above Allocation Request (PAAR) activities was identified for Mozambique that, if implemented, would not only assist southern Mozambique on its path to elimination, but will also reduce the importation of malaria into South Africa. These include LLIN distribution and IRS in Maputo, Gaza and Inhambane provinces in Southern Mozambique. *Source reduction* in South Mozambique was modelled according to these PAAR activities.

Additionally, it was also decided to model two scenarios where interventions are scaled down or stopped entirely to assess the potential impact on malaria transmission. *The Reverse 1 scenario* focused on the potential impact of replacing all IRS conducted with DDT with non-organophosphate or phosphate esters (OPEs) insecticides. A potential outcome is the re-establishment of *Anopheles funestus* into receptive areas (as pyrethroid resistance increases and resistant vector populations move into new areas) resulting in a 2.5-fold increase in transmission (based on historical data from 2000 epidemic) [22–25]. *The Reverse 2 scenario* simulates the impact of targeted IRS efforts to only those places with high vector abundance with the goal to reduce impact of the re-establishment of *Anopheles funestus* into receptive areas. These reverse scenarios are intended to show the hypothetical long-term impact of reducing interventions or financial resources. The two reverse scenarios assume no additional interventions once cases increase.

### Prevention of reintroduction activities

Once the provinces have entered the prevention of reintroduction (POR) phase, it is likely that the intensified activities that lead to elimination will be scaled down and/or changed. Key activities of the malaria programme may also be integrated into existing systems or platforms in the health system, such as IEC through the Community Healthcare Worker (CHW) programme. The following activities were proposed for the POR phase:

- Maintain IRS, larviciding and entomological surveillance (assuming a 5% growth in structures sprayed from 2019 till 2022, after which number of structures sprayed are maintained),
- Continual implementation of active case detection (100% of cases with reactive IRS),
- Maintenance of border units in all provinces, and
- Maintenance of malaria surveillance activities

Moreover, the model assumed that funding and implementation of IEC and training activities will continue to be maintained at current levels and costs will be shared with other health programmes. Costs will be absorbed over a three-year period starting in 2023 and will decrease linearly to 20% of the starting level. As malaria incidence declines, it is likely that IEC will be integrated into existing health promotion activities delivered through national campaigns or through community-based activities (e.g. integrated community health worker programme). It is projected that malaria case management training will be covered by facility-based budgets in the next 5 years.

## Results

### Transmission model predictions

Climate plays an important role in determining the vectoral capacity in an area and malaria incidence is known to vary with changes in rainfall and temperature. All scenarios were modelled under the assumption of low, average and high climate patterns; however, the results presented here are only those from the average climate pattern. Under these scenarios, approximately 33,000 clinical cases with 750 deaths are estimated to be averted nationally between 2018 and 2029 in the Accelerate scenario compared to 50,000 clinical cases and 900 deaths in the Source Reduction scenario.

**Fig. 2:**
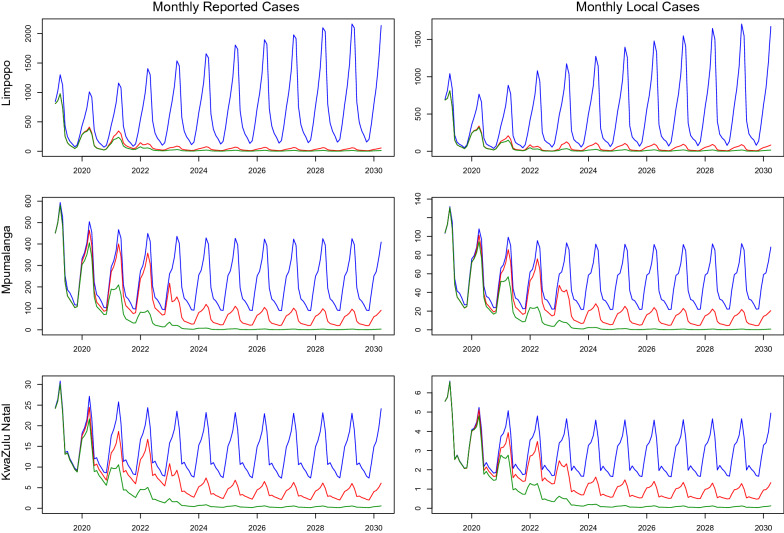
Projected Monthly Malaria Incidence: reported cases (left), local cases (right), by Province for *Business as Usual* (blue), *Accelerate* (red) and *Source Reduction* (green) scenarios

**Fig. 3:**
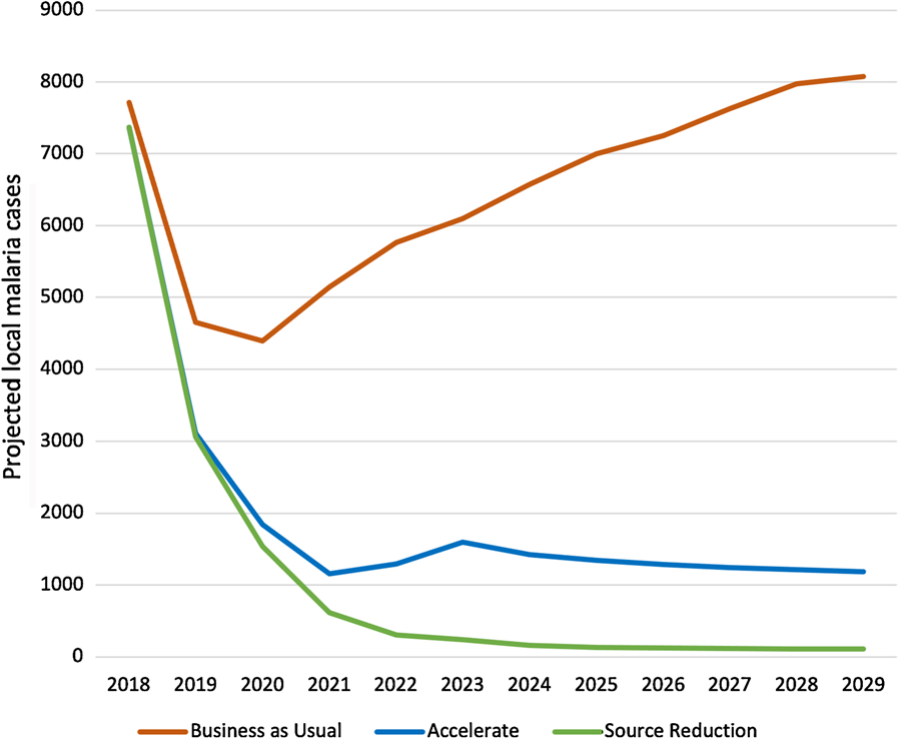
Projected local malaria incidence per year under the three main scenarios: *Business as Usual, Accelerate*, and *Source Reduction* (yellow start indicates projected year of national elimination)

Figure 2 shows monthly malaria cases for the three provinces for the Business as Usual scenario *(blue)*, Accelerate scenario *(red)* and Source Reduction scenario *(green)*. Under the Business as Usual scenario, a large increase in cases is predicted for Limpopo whereas stable incidence is predicted for the other two provinces. Limpopo was, at the time of the study, only spraying half of identified structures, unlike KZN and Mpumalanga, which were close to elimination. Elimination is only predicted to occur in all three provinces under Source Reduction owing to the impact of source reduction in southern Mozambique. Figure 3 shows annual local incidence for the whole country in each of the scenarios with a predicted year of elimination by 2026. Table 4 shows the predicted year with a lower and upper bound of elimination for each of the three endemic provinces. Whenever elimination was not predicted in the model simulation, a value of “ > 2030” was given.

**Table 4:** Predicted year of elimination per province

		Predicted Elimination Year	
Province	Scenario	Mean	Lower and Upper Bound
KwaZulu-Natal	Accelerate	2023	(2023, > 2030)
	Source Reduction	2021	(2021, 2022)
Mpumalanga	Source Reduction	2025	(2025, 2029)
Limpopo	Source Reduction	2026	(2023, > 2030)

### Economic burden of malaria

The model estimated total malaria economic burden under the *Business as Usual, Accelerate, Source Reduction*, and *Reverse scenarios.* For the *Business as Usual* scenario, the total economic burden of malaria in South Africa was estimated at R 3.69 billion (USD 223.3 million) over a 11 period (2018–2029). The economic burden of malaria was estimated at R4.88 billion (USD 295.5 million) and R6.34 billion (~ USD 383.9 million) for the *Accelerate* and *Source Reduction* scenarios, respectively. Table 5 presents national estimates of the economic burden of malaria in South Africa under each scenario.

**Table 5:** Estimates of the economic burden of malaria in South Africa (2018–2029)

Scenarios	Direct Costs to Health System	Direct costs to Individuals	Monetized Indirect costs to society	Total estimated burden
Business as usual	R 3,180,190,344	R 1,132,828,672	R 231,531,993	R 4,544,551,008
Accelerate	R 4,520,328,296	R 137,715,681	R 55,885,400	R 4,713,929,377
Source Reduction	R 5,799,198,770	R 119,350,591	R 39,809,830	R 5,958,359,191
Reverse case scenario	R 23,003,065,946	R 79,643,930,724	R 11,730,138,523	R 114,377,135,193

### Cost projections

**Fig. 4:**
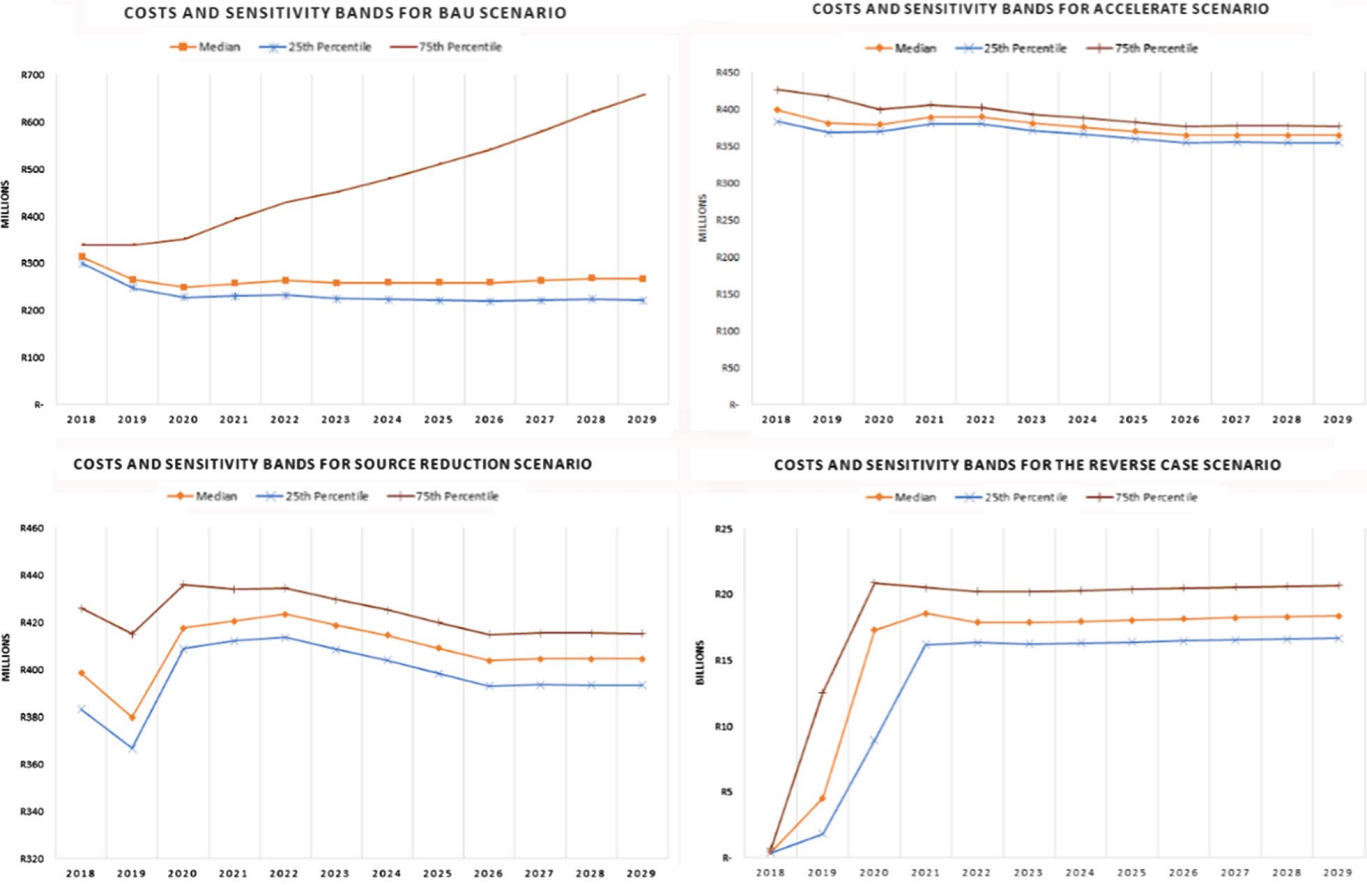
Total Cost of *Business as Usual*, *Accelerate*, *Source Reduction* and *Reverse* case scenarios, 2018–2029

Figure 4 below presents the estimated costs of implementing a package of malaria elimination activities under *Business as Usual*, *Accelerate, Source Reduction* and *Reverse scenarios*. The estimates include the median values for all proposed intervention programme costs and the Inter Quartile Range (IQR) estimates under each of the three strategies plus the reverse case scenario option. Under *Business as Usual* scenario the total costs are estimated to be around R3.41 billion (USD 206.5 million). The costs are projected to increase gradually each year because malaria cases are also expected to increase especially in Limpopo province due to current IRS coverage. Under *Accelerate* and *Source Reduction*, total programme costs are estimated at R4.79 and R5.22 billion, (USD 290.39 and 316.20 million), respectively. The trend for these costs is projected to first increase slightly but will normalize and flatten over time before they start to decline/stabilize in the final years of the programme. Finally, the *Reverse scenario* is projected to increase rapidly in the first two years before achieving stability over the project timeframe. This is expected to increase to over R54.9 billion (USD 3.32 billion) over the 11-year period.

**Fig. 5:**
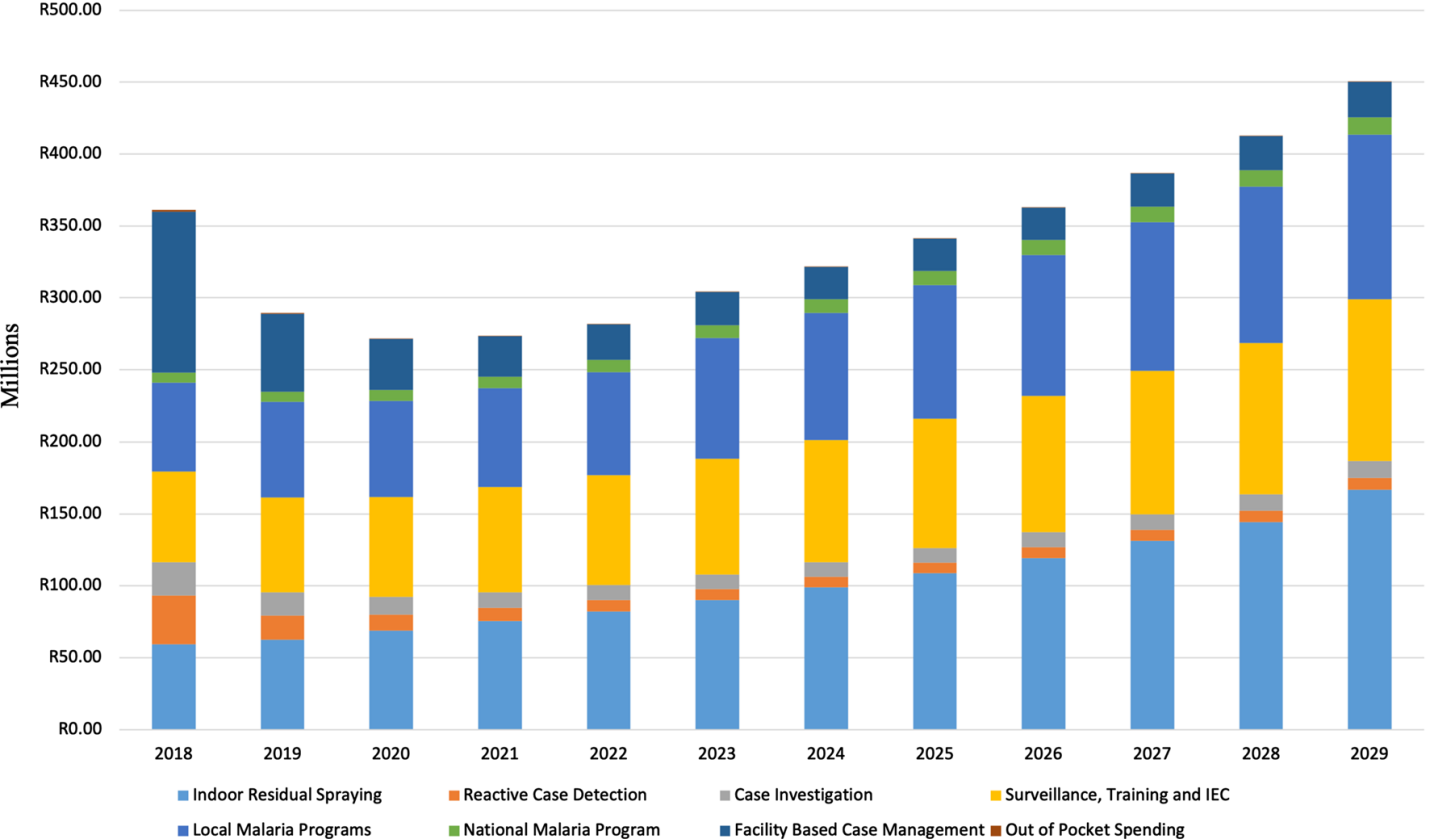
Total Cost per Category for the *Accelerate* Scenario

**Fig. 6:**
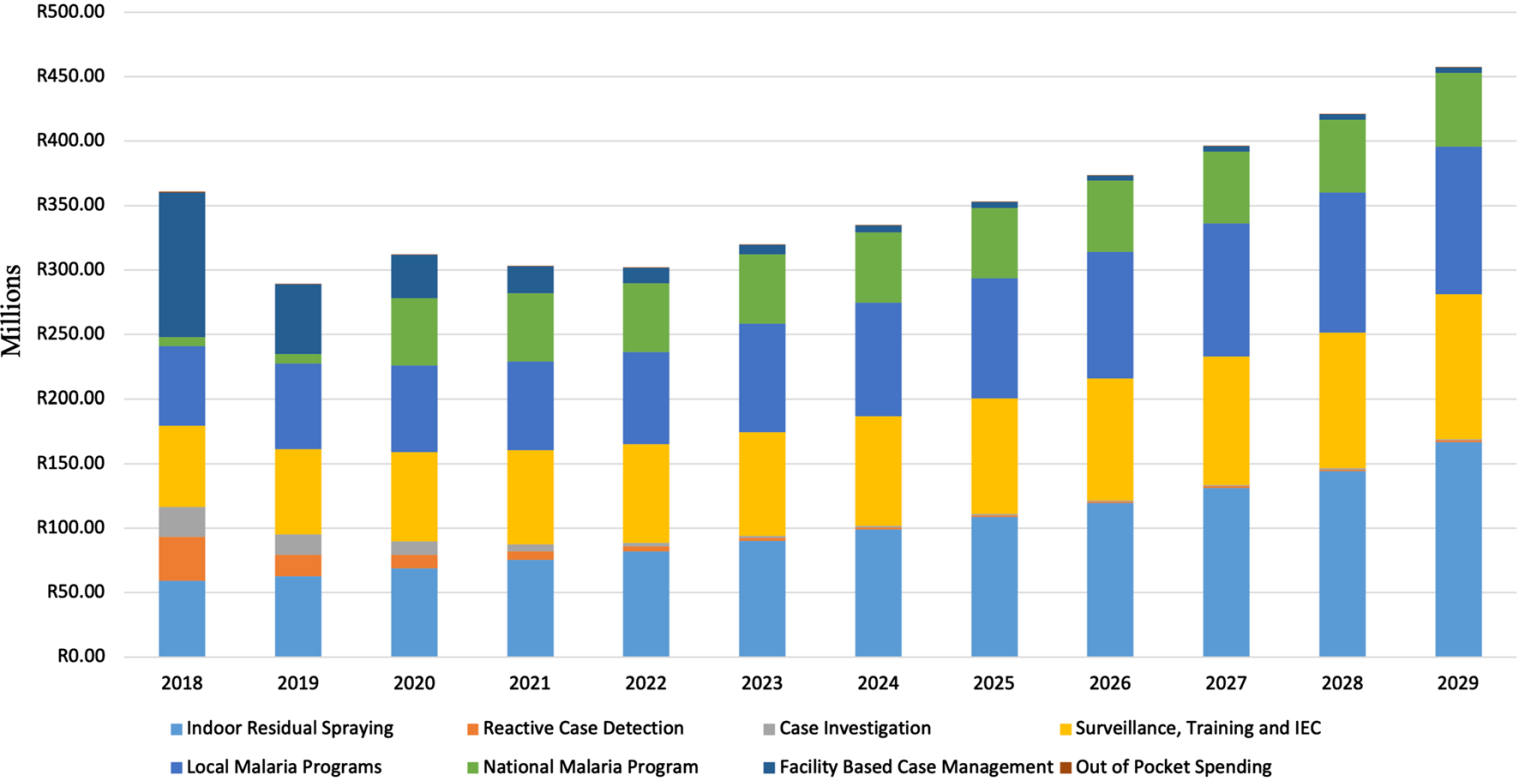
Total Cost per Category for the *Source Reduction* Scenario

In Figs. 5 and 6, the breakdown of costs is presented for the *Accelerate* and *Source Reduction* scenarios respectively. Among others, the activities evaluated include, IRS, Surveillance, Training and IEC materials, Reactive Case Detection, Facility-based Case Management, Out of Pocket expenditures, Provincial and NMCP costs and others. Facility-based case management and case detection activities make up a larger proportion of total expenditure in the first years and decline over time. Programme management costs are relatively constant throughout the implementation phase. Reactive case detection and out of pocket costs for malaria treatment are also projected to decline over time as compared to the initial years of the programme based on the calibration of the model. It is noted that resources to support the NMCP are projected to increase for the *Source Reduction* option to be able to support vector control activities in Mozambique as shown in Fig. 6. Overall, malaria cases were projected to decline over time with total costs are projected to steadily increase at a decreasing rate throughout the elimination period under the scenarios of interest to achieve elimination.

### Benefits of elimination

To determine the return on investment per scenario, the expected benefits from all the interventions for each scenario were added together and then divided by the total costs for each scenario over the 11-year period (2018–2030). Table 6 presents the estimated total ROI for each scenario in comparison with the *Business as usual* scenario. The *Source Reduction* scenario is projected to reduce up to 16,376 additional malaria cases and prevent about 135 more deaths when compared to implementing only the *Accelerate* scenario. The ROI under *Source Reduction* is 4.41 and almost three times higher than the projected ROI under the *Accelerate Scenario* (1.06).

**Table 6:** Costs and benefits of malaria elimination compared to counterfactuals, 2018–2029 (ZAR)

Scenario Comparison	Total Clinical Cases averted	Total Death averted	Net National Benefits (Loss)	Total Net Benefits (Loss)	Total Incremental Costs	SA National ROI	Total ROI (SA plus S. Moz)
*BaU* vs. *Source Reduction*	(49,734)	(900)	R.1,984,548,717	R.3,908,046,782	R.1,719,008,427	4.41	7.42
*BaU* vs. *Accelerate*	(33,358)	(765)	R.1,972,195,299	N/A	R.1,340,137,953	1.06	*N/A*
*BaU* vs. *Reverse*	7,458,555	121,177	(R.68,330,533,536)	N/A	R.182,476,365,815	(5.60)	*N/A*

## Discussion

The relatively high projected cost necessary to achieve malaria elimination in South Africa is a result of several contributing factors. The investment case model assumed that the South African population would continue to experience a 1.1% p.a. growth. Population growth is often cited as a reason to ensure that the malaria programme is funded even after the elimination target is achieved [26]. It was assumed that South Africa would, in line with previous years, continue to experience an influx of migrants from neighbouring countries with high malaria burden. This is conditional on economic opportunities in South Africa continuing to attract migrant labourers as well as economic conditions in neighbouring countries. Moreover, as malaria cases decline towards elimination, resources would be reallocated to sustain malaria surveillance in South Africa. Improvement of malaria surveillance is key to preventing future malaria epidemics. Widespread IRS was modelled to remain an important intervention in South Africa because of the high malaria burden in its neighbouring countries. Finally, some studies have demonstrated total cost reduction during the elimination and POR phases, however, such reductions need to be achieved given the country context and the strength of the local health system to swiftly deal with malaria epidemics should they occur [27]. While countries in isolated small islands like Mauritius and Sri Lanka have been able to reduce total malaria spending during the elimination and POR phases, elimination in South Africa will likely require sustained investments through POR given the major challenge of cross border migration from high malaria burden countries and the resulted influx of imported cases. Of importance to note is the fact that facility-based cost of malaria case management would decline significantly after the 1st year of the proposed programme implementation. The decline in malaria case management is expected because of the expanded rollout of effective malaria prevention activities. Health facilities would therefore not need to purchase and stock large amounts of anti-malarial drugs since both outpatient visits and hospitalization are projected to decline substantially after the *Source Reduction* scenario is implemented.

**Table 7:** Approved Conditional and Co-Financing Resource Allocation for Malaria Elimination. Source: South African National Treasury, 2018

	2019/2020	2020/2021	2021/2022	Total
Conditional grant allocation	R 90,425,000	R 117,198,000	111,188,000	R 318,811,000
Co-financing allocation		R 30,000,000	R30,000,000	R 60,000,000
Grand total				R 378,811,000

Successful malaria elimination in South Africa depends on increased regional resource mobilization efforts from both domestic and external sources. The 2017 malaria resurgence in at least two of the three malaria endemic provinces heightened the need for the development of South Africa’s malaria investment case. The investment case for malaria elimination was a catalyst to better understand how health and broader economic gains could be achieved in South Africa. Given the significant number of imported cases from southern Mozambique, it was argued that adequately financing malaria elimination within South Africa’s borders alone would not be enough to achieve elimination and realize these economic gains. Amidst the recently reports of observed parasite resistance to artemether-lumefantrine drugs in Rwanda, effective case management of both local and imported cases cannot be over-emphasized [28]. Remaining vigilant and deliberate efforts to control imported malaria must be sustained to mitigate the potential spread of drug-resistant malaria parasites within the region, thereby reducing the impact of efforts to eliminate malaria.

Upon completion of the investment case study, the findings were presented to South African government officials to help understand and mobilize additional domestic resources for malaria elimination. The resource mobilization proposal presented Options 1 (*Accelerate scenario*) and 2 (*Source reduction scenario*) with recommendation to create two sustainable financing mechanisms for achieving malaria elimination in South Africa. The South African government approved the proposal to fully fund Source Reduction (*source reduction scenario*) and establish the two new funding mechanisms for malaria.

The evidence generated through this investment case was a useful tool for the government to optimize a mix of interventions and budget requirements necessary to achieve national malaria elimination in South Africa. The technical task team played a critical role by engaging stakeholders and building consensus in the development of a robust malaria budget request. Key government decision makers supported the budget request to fund the scenario to achieve malaria elimination by 2026, which demonstrated that a significant and timely increase in malaria financing could achieve a favorable ROI through reduction of malaria-associated morbidity and mortality.

Informed by the findings of this investment case, the budget request submitted to the government emphasized the need to fund national malaria elimination through the modelled *Source Reduction* scenario (the scale up of interventions in the *Accelerate scenario* and an aggressive approach to reducing imported cases by implementing source reduction activities in southern Mozambique). Following the findings of the investment case, the government of South Africa committed R318.8 million (USD 19.28 million) from 2019/20 to 2021/22 financial years as a conditional grant for the malaria programmes in the provincial health departments as well as full funding of the *Source Reduction* scenario (Table 7). An additional R30 million (~ USD 2.0 million) per annum was allocated towards a co-financing mechanism to support source reduction activities in southern Mozambique. The investment case resulted in an unprecedented increase in political support and domestic resource allocation for the NMCP. For instance, the initiative resulted in an estimated 36% increase in the provincial malaria budgets in 2018/2019 and 2019/2020. To many malaria experts in South Africa, this was unprecedented given the low policy priority malaria control had received locally compared to other diseases considered to be of higher priority like HIV-AIDS, TB and various non-communicable illnesses. The adopted co-financing mechanism is the first of its kind in the region with the government committing substantial domestic funds to support malaria control efforts within South Africa and in neighbouring Mozambique. The process of using the investment case to mobilise domestic resources for malaria elimination is described in Kollipara et al. [31].

### Study limitations

This investment case for malaria control and elimination in South Africa had several limitations. While the best attempts were made to quantify the impact and cost of implementation malaria interventions, the investment case was not able to factor in operational challenges of implementing the set of interventions. Studies have shown that there exist many challenges when implementing malaria elimination interventions to reach targets [29]. In South Africa for instance, a study estimated that most health workers were pessimistic that elimination targets could actually be achieved because the majority of them were not involved in the planning process. [30]. At the time of the study, no standard definition existed for population at risk of malaria at a subnational level and model-specific assumptions were required. The use of districts (admin level 2) as spatial transmission unit of analysis may also be unrealistic due to lack of consistent and comparable data across all three malaria endemic provinces in South Africa. Additionally, it was not always possible to determine the proportion of malaria cases that could be defined as severe, which would significantly impact the inpatient treatment costs.

The effectiveness of the proposed new cross-border malaria screening units depends very much on where they are located and the sustainability of their funding and staffing. With limited data, the effectiveness of border units may only be measured once the programmes are implemented. Finally, the intervention choice and cost estimates for the source reduction strategy in southern Mozambique were determined based on the PAAR activities in the MOSASWA concept note 2018, which was unfunded. The source reduction activities were thus directed by the concept note, rather than through a modelling optimization to determine the best mix of interventions to implement in southern Mozambique. Nevertheless, the South African Investment Case was the first national epidemiological-economic model for malaria in South Africa and the resultant success in securing funding is a considerable step forward towards continued evidence-based decision support on the path to malaria elimination.

## Conclusion

Malaria elimination in South Africa is feasible and economically worthwhile. The findings of this study project that with sustained investment, national elimination is achievable within an 8-year period starting in 2018 to 2026. In addition, national elimination will have a positive ROI and is, therefore, good value for money. The reverse case scenario underscores the importance of political will and sustainable financial commitment to malaria elimination in South Africa. Source reduction in southern Mozambique is a key intervention for South Africa to achieve its ambitious malaria elimination goal. The investment case findings show that the total cost of the malaria programme will first increase slightly and then will remain stable for a period of at least four years. Once elimination is achieved, the costs will decline slightly in response to declining malaria cases and treatment costs, but some costs related to surveillance and programme management will continue beyond elimination. A decline of malaria incidence in neighbouring countries will translate to  reduced malaria importation to South Africa and consequently lower malaria management costs.

South Africa is likely to face many challenges on the path to malaria elimination. The impact of the COVID-19 pandemic on the elimination targets is yet to be determined. The South African Investment Case was an example of leveraging the expert knowledge of the Investment Case Task Team to develop an epidemiological-economic model to determine the costs and benefits of malaria elimination to secure increased domestic funding. This framework is additionally of benefit to other LMIC and countries in the E8 to model and cost paths to elimination and to make the case for financial resource mobilization locally. Through regional collaboration, achieving malaria elimination may be accelerated and sustained.

## Supplementary Information

**Additional file 1. **METC-South Africa: Mathematical model description.

## Data Availability

The datasets used and/or analysed during the current study are available from the  National Department of Health on reasonable request.
