# Supplementary material for: Investment case for malaria elimination in South Africa: a financing model for resource mobilization to accelerate regional malaria elimination
Source: Malar J. 2021 Aug 16;20:344. doi: 10.1186/s12936-021-03875-z (PMC8365569; doi:10.1186/s12936-021-03875-z)
Supplement: Supplementary file 1 — Additional file 1. METC-South Africa: Mathematical model description. [file 12936_2021_3875_MOESM1_ESM.docx]

**METC-South Africa Mathematical model description**

**Sheetal Silal^1,2^ & Lisa White^3^**

^1^ Modelling and Simulation Hub, Africa (MASHA), Department of Statistical Sciences, University of Cape Town, University of Cape Town, Rondebosch, Cape Town 7700, South Africa

^2^ Nuffield Department of Medicine, Centre for Global Health, University of Oxford, Oxford, UK

^3^Big Data Institute, Li Ka Shing Centre for Health Information and Discovery, Nuffield Department of Medicine, University of Oxford, Oxford, UK

This document provides a description of the methodology, equations and parameters underlying the mathematical model for *P. falciparum* malaria transmission in South Africa.

**Malaria Elimination Transmission and Costing (METC)-Country Suite** is a set of mathematical models developed by the Modelling and Simulation Hub, Africa (MASHA, University of Cape Town) and the Oxford Modelling for Global Health group (OMGH, Oxford University) to guide national malaria control and elimination efforts. It is the combination of epidemiological and cost data collection, curation and analysis with multi- and single species, spatially explicit transmission models and simple malaria transmission models. These models may be used to predict both the health outcomes and costs associated with various options for achieving a given malaria elimination strategy.

The METC-Country suite has been adapted to the South African malaria environment to form the **METC-South Africa** model.

The METC-South Africa model is characterised as follows:

Species:

*Plasmodium falciparum*

Methodology:

Compartmental non-linear Ordinary Differential Equation model in a meta-population framework coded in R and C++

Spatial Resolution:

Subnational (District level: South African admin levels are national -> province -> district -> local municipality -> ward (voting))

Owing the changing local municipal borders since 2000 and the unavailabilty of case data by new local municipality boundaries, spatial regions in the model are defined as districts and neighbouring countries:

- - Limpopo - Capricorn, Greater Sekhukhune, Vhembe, Mopani, Waterberg
  - Mpumalanga - Ehlanzeni, Gert Sibande, Nkangala
  - KwaZulu-Natal - uMkhanyakude, Zululand, Uthungulu
  - Southern Mozambique
  - Swaziland
  - Zimbabwe
  - Remaining E8 countries

Supporting data

- Statistics South Africa: Population data, household size, no. of rooms per household
- Malaria Incidence Data: Access to the MIS databases for Limpopo, KwaZulu-Natal and Mpumalanga to determine (where possible) reported incidence, deaths, treatment, access point (facility/hospital), method of detection (active/passive/proactive) and sources of infection by district from 2010 to 2018
- IRS Data:  Access to spray databases for Limpopo, KwaZulu-Natal and Mpumalanga to determine (where possible) the number of structures sprayed, targeted structures, insecticide type (DDT, Pyrethroid) and number of people protected through IRS  by district and spray type from 2010 to 2018
- Climate Data: Climate Hazards Group InfraRed Precipitation with Station data (CHIRPS) 30+ year quasi-global rainfall dataset, curated by MEI at UCSF, and prepared for district municipalities in South Africa from 1998 to 2018.
- Malaria Incidence Data: Access to annual data for Southern Mozambique (Maputo, Gaza and Inhambane) to determine reported incidence and deaths from 2010 to 2018
- Mozambique intervention Data: Access to annual ITN distribution and IRS data and population data for Southern Mozambique (Maputo, Gaza and Inhambane) to determine vector control coverage from 2010 to 2018
- World Malaria Reports: Incidence and intervention data for surrounding Elimination 8 countries.
- Cross border unit data: Access to 3^rd^ party supplier data and E8 data on cross border units in South Africa to determine number of screens and positive cases detected since inception in South Africa from June 2017 to July 2018.
- Cost data for all of the above curated by the Malaria Elimination Initiative, University of California, San Francisco.

*Plasmodium falciparum model*

We use a compartmental model for the transmission of *P. falciparum* malaria. Its structure is similar to previously published models [1-2, 94-98]. There are four infection classes in this model representing infections that are: severe; clinical; asymptomatic and detectable by microscopy; asymptomatic and undetectable by microscopy. Each infection class has a distribution of parasitaemia associated with it that is used to estimate the sensitivity of various diagnostic tests. Each infection class also has an infectiousness associated with it based on infectivity data. The probability of individuals entering each class of infection is dependent on their immunity status. We assume that untreated individuals will transition from higher to lower severity infection classes as they recover and that they can be boosted to higher severity classes on superinfection. We assume that treated individuals test positive for HRP2 after clearance of asexual parasitaemia for different durations depending on the detection limit of the test used.

The system is depicted in Figure 1 and described by the following set of ordinary differential equations with compartment descriptions in Table 1:

where eln is the Climate Hazards Group InfraRed Precipitation with Station data (CHIRPS) 30+ year quasi-global rainfall index time series, standardised between 0 and 1 and smoothed with a running median to estimate effect size. (Accessible at: https://www.chc.ucsb.edu/data/chirps).

Figure *Plasmodium falciparum* model flow diagram

Table 1 Model Variables

| Symbol | Definition |
| --- | --- |
| *Falciparum* Variables | |
| S | Uninfected and non-immune population |
| H | Uninfected and immune population who test positive by RDT |
| R | Uninfected and immune population |
| I_N_ | Infected and asymptomatic malaria population undetectable by microscopy |
| I_A_ | Infected and asymptomatic malaria population detectable by microscopy |
| I_C_ | Infected and clinical malaria population |
| I_S_ | Infected and severe malaria population |
| T_0_ | Population under effective treatment by other means (E.g. Private care) |
| T_V_ | Population under effective treatment by Village Malaria Worker |
| T_H_ | Population under effective treatment through Health Information System |

Table 2 Model Parameters

| Symbol | Definition | Value | Units | Sim Range | | Source |
| --- | --- | --- | --- | --- | --- | --- |
| Common Parameters | | | | | | |
| *φ* | Month of peak transmission | 1 | month |  | data | |
| *a* | Amplitude of seasonal variation | 1 | na | (0,1) |  | |
| δ_m_ | Average life expectancy of mosquito | 14 | days | (10,20) | 19,24 | |
| b | Number of mosquito bites per human per day | 1/3 | day^-1^ | (0.1,0.5) | 23,34 | |
| p_exo | Proportion of bites that occur outdoors | 2/3 | % | (0.6, 0.8) | 91,92 | |
| ε_m_ | Probability that a bite from an infectious mosquito will result in infection | 50 | % | (20,50) | 23, 48, 50 | |
| ε_h_ | Probability that a bite from an infectious human will result in infection | 50 | % | (7,64) | 20,25 | |
| 1/γ_M_ | Duration of latent period in mosquitoes | 10 | days | (5, 15) | 11, 20-22,24, 51 | |
| Falciparum Parameters | | | | | | |
| *p_S_* | Proportion of non-immune individuals expected to develop clinical malaria after infection | 95 | % | (90,100) | | 10, 11 |
| *p_R_* | Proportion of immune individuals expected to develop clinical malaria after infection | 10 | % | (0,77) | | 12 |
| *p_SN_* | Proportion of non-immune individuals expected to develop sub-patent infection upon challenge | 10 | % | (0,20) | |  |
| *p_RN_* | Proportion of immune individuals expected to develop sub-patent infection upon challenge | 50 | % | (30,70) | |  |
| 1/r _S_ | Duration of symptoms in an untreated severe infection | 5 | day | (1,10) | |  |
| 1/* r* _C_ | Duration of symptoms in an untreated clinical infection | 10 | days | (1,15) | | 13, 14 |
| 1/r _A_ | Duration of symptoms in an untreated asymptomatic infection | 130 | days | (60, 200) | | 16,30,31 |
| *τ* _SEV_ | Proportion of severe malaria that is treated | 95 | % | (80, 100) | |  |
| *p_sev_* | Proportion of clinical infections that become severe | 3 | % | (5,25) | | 15,32 |
| **_A_ | Relative infectiousness of asymptomatic infection compared to clinical infection | 12.6/27 | na | (0,0.50) | | 18 |
| **_N_ | Relative infectiousness of sub-patent infection compared to clinical infection | 3.9/27 | na | (0., 0.25) | | 17 |
| **_T_ | Relative infectiousness of treated infections compared to untreated clinical infection | 0.04 | na | (0., 0.25) | | Unpublished study |
| 1/*ω* | Duration of immunity in an individual without challenge | 1 | year | (0.5,10) | | 16 |
| θ_1_ | Probability that untreated severe malaria progresses to death | 80 | % | (70,100) | | 15 |
| θ_2_ | Probability that treated severe malaria progresses to death (quinine)  Probability that treated severe malaria progresses to death (IV artesunate) | 22  15 | % | (15,30)  (10, 40) | | 80-82 |
| 1/γ_H_ | Incubation period and time to gametocytemia in humans | 21 | days | (14,24) | | 11, 20-22, 61 |
| *1/χ* | Period of HRP2 detectability by RDT | 28 | days | (21,37) | | 27-29 |
| *1/r_T_* | Time taken to clear asexual parasites and gametocytes after treatment with ACT | 10 | day | (10,17) | |  |
| *1/r_Q_* | Recovery time with quinine for severe infections | 6 | days | (4,8) | | [38] |
| eff_IRS_ | Efficacy of indoor residual spraying | 38 | % | (25,45) | | [93] |
| eff_HIS_ | Pr(treatment seeking)*Pr(Diagnosis)*Pr(receive treatment) |  | % |  | | estimated from data |
|  | Proportion of infections seeking treatment | 95% | % | (90, 97) | | data |
|  | Proportion of cases receiving diagnosis | 100 | % |  | | data |
|  | Proportion of diagnosed cases receiving treatment | 100 | % |  | | data |
| 1/*μ* | Average life expectancy of the population | 62 | year | (58,66) | | 33 |
| *ptf* | Baseline probability of treatment failure on ACT | 5 | % | (1,10) | | assumption |
| *ptfc* | Probability of being clinical after treatment failure | 0.75 | % | (0.5, 0.9) | | assumption |
| *ptftr* | Probability of seeking trt if clinical, after treatment failure | 0.27 | % | (0.1, 0.4) | | assumption |

**Sub-patent infection and diagnostics**

We assume that parasitaemia (parasites per μl) within each infection class (sub-patent, asymptomatic and clinical) is log-normally distributed as described in [39]. We also use a mixture model approach to obtain the distribution for severe infection using the data from [40].

The following table summarises the model parameters and their sources:

| Description | Unit | Pf Value | Ref | Pv Value | Ref |
| --- | --- | --- | --- | --- | --- |
| Geometric mean parasitaemia for sub-patent infections (mn_N_) | μl^-1^ | 5 | [41] | 5 | [41, 42] |
| Geometric mean parasitaemia for asymptomatic infections(mn_A_) | μl^-1^ | 5158 | [41] | 750 |  |
| Geometric mean parasitaemia for clinical infections(mn_C_) | μl^-1^ | 25000 | [40, 43] | 5000 | [44] |
| Geometric mean parasitaemia for severe infections(mn_S_) | μl^-1^ | 350000 | [40] | 20000 | [45] |
| Log standard deviation of log-normal parasite distribution for sub-patent infections | - | 0.75 |  | 0.75 |  |
| Log standard deviation of log-normal parasite distribution for asymptomatic infections | - | 1.5 | [43, 46] | 1.5 | [40, 43] |
| Log standard deviation of log-normal parasite distribution for clinical infections | - | 1.3 | [40, 43] | 1.3 | [8, 40] |
| Log standard deviation of log-normal parasite distribution for severe infections | - | 0.26 | [40] | 4 | [8] |

The following table describes the detection limits also described in [39]:

| Description | Units | Pf Value | Ref |
| --- | --- | --- | --- |
| Detection limit for conventional RDT | μl^-1^ | 200 | [47] |
| Detection limit for microscopy | μl^-1^ | 100 | [47] |
| Detection limit for proposed RDT | μl^-1^ | 5 | [48, 49] |
| Detection limit for conventional qPCR | μl^-1^ | 0.2 | [50] |

Test sensitivity:

The parameters above are used to compute diagnostic sensitivity. For each disease class, i, the sensitivity of a test, x, with detection limit, d_T_, is given by the formula:

$${sens}_{i,x}=1-\frac{1}{2}\left[ 1+erf\left( \frac{d_{T}-\mu_{i}}{\sigma_{i}\sqrt[2]{2}} \right) \right]$$

Where μ_i_ and σ_i_ are the log-mean and the log-standard deviation of the log-normal distribution of parasitaemia for disease class i ε {sub-patent, asymptomatic, clinical, severe).

Test specificity:

It has been shown that treated individuals remain positive by conventional RDT for approximately 28 days after successful clearance of asexual parasites [34-36]. An H compartment (individuals recently recovered who are not infected but test positive by RDT) has therefore been included in the model in order to simulate this. The duration of time spent in the H compartment is dependent on the sensitivity of the RDT to detect HRP2 which is assumed to be linearly correlated with its asexual parasite detection limit.

Duration in each infection class:

For severe, clinical and asymptomatic infection the duration of infection is well documented. For sub-patent infection, we assume that the duration of sub-patent infection, δ_N_, can be extrapolated from the duration of infection of asymptomatic infection, δ_A_, and an assumption of log-linear decline in parasitaemia using the following formula:

$$\delta_{N}=\delta_{A}\frac{\mu_{N}-d_{0}}{\mu_{A}-\mu_{N}}$$

Where μ_N_ is the log-mean of the log-normal distribution of parasitaemia for sub-patent infection, μ_A_ is the log-mean of the log-normal distribution of parasitaemia for asymptomatic infection and d_0_ is the detection limit of the most sensitive test (qPCR).

Using the parameters above, we would expect sub-patent infection to be detectable by qPCR for 75 days.

***Force of infection and Seasonality***

The force of infection on humans, λ is derived by assuming that mosquito dynamics of an SEI model are at a steady state resulting in the following:

where eln is the Climate Hazards Group InfraRed Precipitation with Station data (CHIRPS) 30+ year quasi-global rainfall index time series, standardised between 0 and 1 and smoothed with a running median to estimate effect size. (Accessible at: https://www.chc.ucsb.edu/data/chirps).

***Model Interventions***

The table below summarises the impact that each of the interventions modelled has on model parameters/equations.

| Intervention | Description | Model Impact |
| --- | --- | --- |
| Passive treatment | Treatment probabilities ($\tau)$ for different avenues of treatment (v, h, o) dependent on coverage (cov), treatment-seeking and treatment effectiveness (*eff*) and diagnostic sensitivity (*sens*) | See below |
| $\tau_{v}={cov}_{v}{\times eff}_{v}\times{sens}_{v}$  $\tau_{h}={(1-cov}_{v}{eff}_{v})\times{eff}_{h}\times{sens}_{h}$ $\tau_{o}={(1-cov}_{v}{eff}_{v}- {(1-cov}_{v}{eff}_{v}){eff}_{h})\times{eff}_{o}\times{sens}_{o}$ | | |
| Indoor residual spraying | Number of people protected by IRS as a proportion of the population at risk (*irs*) and the half-life of the insectide (*hlspray*) are used to compute cumulative coverage (irscov). This, together with ability to prevent transmission (*irseff*) is used to decrease the transmission function $\lambda$ | See below |
| $irscov_{t}=irs_{t}+0.5irscov_{t-1}e^{-\frac{1}{12}/(hlspray)}$  $\lambda_{t}^{*}=\left( 1-irscov_{t}\times irseff \right)\times\lambda_{t}$ | | |
| Injectable artesunate | Switching from treatment of severe infections with quinine to injectable artesunate | Parameters decreased:  1/r_Q_ – recovery time  pmort – probability of death of treated severe infections |
| Reactive Case Detection | Reactive detection of sample of population in contact with index indigenous case. Cases are detected based on sensitivity of RDT and level of parasitaemia.  tauRCD: rate of detecting cases | Parameters affected  cov_RCD - programme data  sample- programme data |
| $tauRCD=\left( cov_{RCD}*incidence\left( 1+\frac{1.5sample}{pop} \right) \right)*RDTsensitivity$ | | |
| Cross border surveillance/Proactive Case Detection | Active detection and treatment of screening target per day. Cases are detected based on sensitivity of RDT and level of parasitaemia.  tauproACD: rate of detecting cases | Parameters affected:  cov_proACD- programme data  screens- programme data |
| $tauproRCD=\left( cov_{proACD}*\left( \frac{screens}{importations} \right) \right)*RDTsensitivity$ | | |

***References***

1. White, L.J.*, et al.* The role of simple mathematical models in malaria elimination strategy design. *Malaria journal* **8**, 212 (2009).

2. Silal, S.P., Little, F., Barnes, K.I. & White, L.J. Predicting the impact of border control on malaria transmission: A Simulated Focal Screen and Treat campaign. *In Preparation* (2015).

3. Hendriksen, I.C.*, et al.* Defining falciparum-malaria-attributable severe febrile illness in moderate-to-high transmission settings on the basis of plasma PfHRP2 concentration. *The Journal of infectious diseases* **207**, 351-361 (2013).

4. Mosha, J.F.*, et al.* Epidemiology of subpatent Plasmodium falciparum infection: implications for detection of hotspots with imperfect diagnostics. *Malaria journal* **12**, 221 (2013).

5. Zaloumis, S.*, et al.* Assessing the utility of an anti-malarial pharmacokinetic-pharmacodynamic model for aiding drug clinical development. *Malaria journal* **11**, 303 (2012).

6. Starzengruber, P.*, et al.* High prevalence of asymptomatic malaria in south-eastern Bangladesh. *Malaria journal* **13**, 16 (2014).

7. Grueninger, H. & Hamed, K. Transitioning from malaria control to elimination: the vital role of ACTs. *Trends in parasitology* **29**, 60-64 (2013).

8. Hopkins, H.*, et al.* Highly sensitive detection of malaria parasitemia in a malaria-endemic setting: performance of a new loop-mediated isothermal amplification kit in a remote clinic in Uganda. *The Journal of infectious diseases* **208**, 645-652 (2013).

9. Polley, S.D.*, et al.* Clinical evaluation of a loop-mediated amplification kit for diagnosis of imported malaria. *The Journal of infectious diseases* **208**, 637-644 (2013).

1. Griffin JT, Ferguson NM, Ghani AC: Estimates of the changing age-burden of Plasmodium falciparum malaria disease in sub-Saharan Africa. Nat Commun 2014, 5.
2. Collins, W. E., & Jeffery, G. M. (1999). A retrospective examination of sporozoite-and trophozoite-induced infections with Plasmodium falciparum: development of parasitologic and clinical immunity during primary infection. The American journal of tropical medicine and hygiene, 61(1 Supplement), 4-19.
3. Collins, W. E., & Jeffery, G. M. (1999). A retrospective examination of secondary sporozoite-and trophozoite-induced infections with Plasmodium falciparum: development of parasitologic and clinical immunity following secondary infection. The American journal of tropical medicine and hygiene, 61(1 suppl), 20-35.
4. Griffin JT, Hollingsworth TD, Okell LC, Churcher TS, White M, et al. (2010) Reducing Plasmodium falciparum Malaria Transmission in Africa: A Model-Based Evaluation of Intervention Strategies. PLoS Med 7(8): e1000324. doi: 10.1371/journal.pmed.1000324
5. Miller, M.J., Observations on the natural history of malaria in the semi-resistant West African. Trans R Soc Trop Med Hyg, 1958. 52(2): p. 152-68.
6. Lubell Y, Staedke SG, Greenwood BM, Kamya MR, Molyneux M, et al. (2011) Likely Health Outcomes for Untreated Acute Febrile Illness in the Tropics in Decision and Economic Models; A Delphi Survey. PLoS ONE 6(2): e17439. doi: 10.1371/journal.pone.0017439
7. Filipe JAN, Riley EM, Drakeley CJ, Sutherland CJ, Ghani AC (2007) Determination of the Processes Driving the Acquisition of Immunity to Malaria Using a Mathematical Transmission Model. PLoS Comput Biol 3(12): e255. doi: 10.1371/journal.pcbi.0030255
8. Okell, L. C., Bousema, T., Griffin, J. T., Ouédraogo, A. L., Ghani, A. C., & Drakeley, C. J. (2012). Factors determining the occurrence of submicroscopic malaria infections and their relevance for control. Nature communications, 3, 1237.
9. Kim A Lindblade, Laura Steinhardt, Aaron Samuels, S Patrick Kachur & Laurence Slutsker (2013) The silent threat: asymptomatic parasitemia and malaria transmission, Expert Review of Anti-infective Therapy, 11:6, 623-639
10. Wanji, S., Tanke, T., Atanga, S. N., Ajonina, C., Nicholas, T., & Fontenille, D. (2003). Anopheles species of the mount Cameroon region: biting habits, feeding behaviour and entomological inoculation rates. Tropical Medicine & International Health, 8(7), 643-649.
11. Chitnis, N., Hyman, J. M., & Cushing, J. M. (2008). Determining important parameters in the spread of malaria through the sensitivity analysis of a mathematical model. Bulletin of mathematical biology, 70(5), 1272-1296.
12. Thomson D (1911) A research into the production, life and death of crescents in malignant tertian malaria, in treated and untreated cases, by an enumerative method; the leucocytes in malarial fever: a method of diagnosing malaria long after it is apparently cured. University Press
13. Eyles DE, Young MD (1951) The duration of untreated or inadequately treated Plasmodium falciparum infections in the human host. J Natl Malar Soc 10(4):327–336. Available from
14. Ross R: Some a priori pathometric equations. Br Med J 1915, 1:546-447.
15. Anderson RM, May RM: Infectious diseases of humans: dynamics and control London: Oxford University Press; 1991.
16. Macdonald G: The epidemiology and control of malaria London: Oxford  University Press; 1957.
17. Imwong, M., Stepniewska, K., Tripura, R., Peto, T. J., Lwin, K. M., Vihokhern, B., ... & Keereecharoen, L. (2015). The numerical distributions of parasite densities in asymptomatic malaria. Journal of Infectious Diseases, jiv596.
18. Kyabayinze DJ, Tibenderana JK, Odong GW, Rwakimari JB, Counihan H: Operational accuracy and comparative persistent antigenicity of HRP2 rapid diagnostic tests for Plasmodium falciparum malaria in a hyperendemic region of Uganda. Malar J 2008, 7:221.
19. Swarthout TD, Counihan H, Senga RKK, van den Broek I: Paracheck-Pf accuracy and recently treated Plasmodium falciparum infections: is there a risk of over-diagnosis? Malar J 2007, 6:58
20. Aydin-Schmidt, B., Mubi, M., Morris, U., Petzold, M., Ngasala, B. E., Premji, Z., & Mårtensson, A. (2013). Usefulness of Plasmodium falciparum-specific rapid diagnostic tests for assessment of parasite clearance and detection of recurrent infections after artemisinin-based combination therapy. Malaria journal, 12(1), 349.
21. Felger I, Maire M, Bretscher MT, Falk N, Tiaden A, et al. (2012) The Dynamics of Natural Plasmodium falciparum Infections. PLoS ONE 7(9): e45542. doi: 10.1371/journal.pone.0045542
22. Sama, W., Owusu-Agyei, S., Felger, I., Vounatsou, P. and Smith, T. (2005), An immigration–death model to estimate the duration of malaria infection when detectability of the parasite is imperfect. Statist. Med., 24: 3269–3288. doi:10.1002/sim.2189
23. Jamie T. Griffin, T. Déirdre Hollingsworth, Hugh Reyburn, Chris J. Drakeley, Eleanor M. Riley, Azra C. Ghani. (2015) Gradual acquisition of immunity to severe malaria with increasing exposure Proc. R. Soc. B 2015 282 20142657; DOI: 10.1098/rspb.2014.2657. Published 7 January 2015
24. Statistics South Africa, Mid-year Population Estimates 2017, Available at: <http://www.statssa.gov.za/publications/P0302/P03022017.pdf> . Accessed: 1 May 2018
25. Churcher, T. S., Trape, J. F., & Cohuet, A. (2015). Human-to-mosquito transmission efficiency increases as malaria is controlled. Nature communications, 6.
26. Center for Disease Control. Available at: <http://www.cdc.gov/malaria/malaria_worldwide/reduction/itn.html> Accessed: 20 January 2015
27. Trampuz A, Jereb M, Muzlovic I, Prabhu RM. Clinical review: Severe malaria. Critical Care. 2003;7(4):315-323.
28. High-Throughput Ultrasensitive Molecular Techniques for Quantifying Low-Density Malaria Parasitemias
29. Makanga M, Krudsood S: The clinical efficacy of artemether/lumefantrine (Coartem). Malar J 2009, 8 Suppl 1:S5, [http://www.malariajournal.com/content/8/S1/S5].
30. Are ́valo-Herrera M, Forero-Pen ̃a DA, Rubiano K, Go ́mez-Hincapie J, Mart ́ınez NL, et al. (2014) Plasmodium vivax Sporozoite Challenge in Malaria-Na ̈ıve and Semi-Immune Colombian Volunteers. PLOS ONE 9(6): e99754. doi:10.1371/journal.pone.0099754
31. Cheng Q, Cunningham J, Gatton ML (2015) Systematic Review of Sub-microscopic P. vivax Infections: Prevalence and Determining Factors. PLoS Negl Trop Dis 9(1): e3413. doi:10.1371/journal.pntd.0003413
32. Harris, I., Sharrock, W. W., Bain, L. M., Gray, K. A., Bobogare, A., Boaz, L., ... & Gatton, M. L. (2010). A large proportion of asymptomatic Plasmodium infections with low and sub-microscopic parasite densities in the low transmission setting of Temotu Province, Solomon Islands: challenges for malaria diagnostics in an elimination setting. Malar J, 9(254), 10-1186.
33. Yount Jr, E. H., & Coggeshall, L. T. (1949). Status of immunity following cure of recurrent vivax malaria. American Journal of Tropical Medicine, 29(5), 701-5.
34. Markell, E. K., & Voge, M. (1965). Medical Parasitology. Academic Medicine, 40(7), 719.
35. Adolphe, M. (Ed.). (2013). Chemotherapy: Proceedings of the 7th International Congress of Pharmacology, Paris, 1978 (Vol. 10). Elsevier.
36. Naing C, Whittaker MA, Nyunt Wai V, Mak JW (2014) Is Plasmodium vivax Malaria a Severe Malaria?: A Systematic Review and Meta-Analysis. PLoS Negl Trop Dis 8(8): e3071. doi:10.1371/journal.pntd.0003071
37. Vallejo, A. F., García, J., Amado-Garavito, A. B., Arévalo-Herrera, M., & Herrera, S. (2016). Plasmodium vivax gametocyte infectivity in sub-microscopic infections. Malaria journal, 15(1), 1.
38. Rahimi, B. A., Thakkinstian, A., White, N. J., Sirivichayakul, C., Dondorp, A. M., & Chokejindachai, W. (2014). Severe vivax malaria: a systematic review and meta-analysis of clinical studies since 1900. Malaria J, 13, 481.
39. Robinson LJ, Wampfler R, Betuela I, Karl S, White MT, Li Wai Suen CSN, et al. (2015) Strategies for Understanding and Reducing the Plasmodium vivax and Plasmodium ovale Hypnozoite Reservoir in Papua New Guinean Children: A Randomised Placebo-Controlled Trial and Mathematical Model. PLoS Med 12(10): e1001891. doi:10.1371/journal. pmed.1001891
40. Bharti AR, Chuquiyauri R, Brouwer KC, Stancil J, Lin J, Llanos-cuentas A, et al. Experimental infection of the neotropical malaria vector Anopheles darlingi by human patient-derived Plasmodium vivax in the Peruvian Amazon. Am J Trop Med Hyg. 2007;75: 610–616.
41. Smith DL, Drakeley CJ, Chiyaka C, Hay SI. A quantitative analysis of transmission efficiency versus intensity for malaria. Nat Commun. 2010;1: 108. doi:10.1038/ncomms1107
42. Chamchod, F., & Beier, J. C. (2013). Modeling Plasmodium vivax: relapses, treatment, seasonality, and G6PD deficiency. Journal of theoretical biology, 316, 25-34.
43. Baird, J. K., & Hoffman, S. L. (2004). Primaquine therapy for malaria. Clinical infectious diseases, 39(9), 1336-1345.
44. White, N. J. (2008). The role of anti-malarial drugs in eliminating malaria. Malaria journal, 7(1), 1.
45. White, M. T., Karl, S., Battle, K. E., Hay, S. I., Mueller, I., & Ghani, A. C. (2014). Modelling the contribution of the hypnozoite reservoir to Plasmodium vivax transmission. Elife, 3, e04692.
46. Louicharoen, C., Patin, E., Paul, R., Nuchprayoon, I., Witoonpanich, B., Peerapittayamongkol, C., ... & Quintana-Murci, L. (2009). Positively selected G6PD-Mahidol mutation reduces Plasmodium vivax density in Southeast Asians. Science, 326(5959), 1546-1549.
47. Howes RE, Piel FB, Patil AP, Nyangiri OA, Gething PW, et al. (2012) G6PD Deficiency Prevalence and Estimates of Affected Populations in Malaria Endemic Countries: A Geostatistical Model-Based Map. PLoS Med 9(11): e1001339. doi:10.1371/journal.pmed.1001339
48. Lin JT, Bethell D, Tyner SD, Lon C, Shah NK, et al. (2011) Plasmodium falciparum Gametocyte Carriage Is Associated with Subsequent Plasmodium vivax Relapse after Treatment. PLoS ONE 6(4): e18716. doi:10.1371/journal.pone.0018716
49. Snounou, G., & White, N. J. (2004). The co-existence of Plasmodium: sidelights from falciparum and vivax malaria in Thailand. Trends in parasitology, 20(7), 333-339.
50. White, N. J. (2011). Determinants of relapse periodicity in Plasmodium vivax malaria. Malaria journal, 10(1), 1-36.
51. Douglas, N. M., Nosten, F., Ashley, E. A., Phaiphun, L., van Vugt, M., Singhasivanon, P., ... & Price, R. N. (2011). Plasmodium vivax recurrence following falciparum and mixed species malaria: risk factors and effect of antimalarial kinetics. Clinical infectious diseases, 52(5), 612-620.
52. Bousema, T., & Drakeley, C. (2011). Epidemiology and infectivity of Plasmodium falciparum and Plasmodium vivax gametocytes in relation to malaria control and elimination. Clinical microbiology reviews, 24(2), 377-410.
53. Brasil, P., de Pina Costa, A., Pedro, R. S., da Silveira Bressan, C., da Silva, S., Tauil, P. L., & Daniel-Ribeiro, C. T. (2011). Unexpectedly long incubation period of Plasmodium vivax malaria, in the absence of chemoprophylaxis, in patients diagnosed outside the transmission area in Brazil. Malar J, 10, 122.
54. Pereira, E. A., Ishikawa, E. A., & Fontes, C. J. (2011). Adherence to Plasmodium vivax malaria treatment in the Brazilian Amazon Region. Malar J, 10, 355.
55. Cheoymang, A., Ruenweerayut, R., Muhamad, P., Rungsihirunrat, K., & Na-Bangchang, K. (2015). Patients’ adherence and clinical effectiveness of a 14-day course of primaquine when given with a 3-day chloroquine in patients with Plasmodium vivax at the Thai–Myanmar border. Acta tropica, 152, 151-156.
56. Leslie, T., Rab, M. A., Ahmadzai, H., Durrani, N., Fayaz, M., Kolaczinski, J., & Rowland, M. (2004). Compliance with 14-day primaquine therapy for radical cure of vivax malaria—a randomized placebo-controlled trial comparing unsupervised with supervised treatment. Transactions of the Royal Society of Tropical Medicine and Hygiene, 98(3), 168-173.
57. World Health Organisation (2014) Evidence Review Group meeting report, “Point-of-care G6PD testing to support safe use of primaquine for the treatment of vivax malaria” , Available from: <http://www.who.int/malaria/mpac/mpac-march2015-erg-g6pd.pdf> Accessed on 23 May 2016
58. Abba, K., Kirkham, A. J., Olliaro, P. L., Deeks, J. J., Donegan, S., Garner, P., & Takwoingi, Y. (2014). Rapid diagnostic tests for diagnosing uncomplicated non-falciparum or Plasmodium vivax malaria in endemic countries. Cochrane Database Syst Rev, 12.
59. Mayxay, M., Pukrittayakamee, S., Newton, P. N., & White, N. J. (2004). Mixed-species malaria infections in humans. Trends in parasitology, 20(5), 233-240.
60. Mayxay, M. et al. (2001) Identification of cryptic coinfection with Plasmodium falciparum in patients presenting with vivax malaria. Am. J. Trop. Med. Hyg. 65, 588–592
61. Maitland, K. et al. (1997) Plasmodium vivax and P. falciparum: biological interactions and the possibility of cross-species immunity. Parasitol. Today 13, 227–231
62. Jeffery,G.M.(1966) Epidemiological significance of repeated infections with homologous and heterologous strains and species of Plasmodium. Bull. World Health Organ. 35, 873–882
63. Guindo A, Fairhurst RM, Doumbo OK, Wellems TE, Diallo DA (2007) X- linked G6PD deficiency protects hemizygous males but not heterozygous females against severe malaria. PLoS Med 4: e66. doi:10.1371/journal.pmed.0040066
64. Ruwende C, Khoo SC, Snow RW, Yates SN, Kwiatkowski D, et al. (1995) Natural selection of hemi- and heterozygotes for G6PD deficiency in Africa by resistance to severe malaria. Nature 376: 246–249.
65. Leslie T, Briceno M, Mayan I, Mohammed N, Klinkenberg E, et al. (2010) The impact of phenotypic and genotypic G6PD deficiency on risk of Plasmodium vivax infection: a case-control study amongst Afghan refugees in Pakistan. PLoS Med 7: e1000283. doi:10.1371/journal.pmed.1000283
66. Hedrick PW (2011) Population genetics of malaria resistance in humans. Heredity (Edinb) 107: 283–304.
67. Luzzatto L (2012) G6PD deficiency and malaria selection. Heredity (Edinb) 108: 456.
68. Pasvol, G. (2005). The treatment of complicated and severe malaria. British medical bulletin, 75(1), 29-47.
69. Gething PW, Van Boeckel TP, Smith DL, Guerra CA, Patil AP, Snow RW, Hay SI. (2011). Modelling the global constraints of temperature on transmission of Plasmodium falciparum and P. vivax. Parasites and Vectors 4:92. doi: 10.1186/1756-3305-4-92.
70. Cappellini MD, Fiorelli G. Glucose-6-phosphate dehydrogenase deficiency. Lancet. 2008; 371:64– 74. [PubMed: 18177777]
71. Guidelines for the Treatment of Malaria. 3rd edition. Geneva: World Health Organization; 2015. 7, TREATMENT OF SEVERE MALARIA. Available from: <https://www.ncbi.nlm.nih.gov/books/NBK294445/>
72. Achan, J., Talisuna, A. O., Erhart, A., Yeka, A., Tibenderana, J. K., Baliraine, F. N., ... & D'Alessandro, U. (2011). Quinine, an old anti-malarial drug in a modern world: role in the treatment of malaria. Malaria journal, 10(1), 1.
73. Dondorp A, Nosten F, Stepniewska K, Day N, White N: Artesunate versus quinine for treatment of severe falciparum malaria: a randomised trial. Lancet. 2005, 366 (9487): 717-725.
74. Price RN, Douglas NM & Anstey NM (2009) New develop- ments in Plasmodium vivax malaria: severe disease and the rise of chloroquine resistance. Current Opinion in Infectious Diseases 22, 430–435.
75. Tjitra E, Anstey NM & Sugiarto P (2008) Multidrug-resistant Plasmodium vivax associated with severe and fatal malaria: a prospective study in Papua, Indonesia. PLoS Medicine 5, e128.
76. Barcus MJ, Basri H, Picarima H, et al. (2007) Demographic risk factors for severe and fatal vivax and falciparum malaria among hospital admissions in northeastern Indonesian Papua. Ameri- can Journal of Tropical Medicine and Hygiene 77, 984–991.
77. Takeuchi, R., Lawpoolsri, S., Imwong, M., Kobayashi, J., Kaewkungwal, J., Pukrittayakamee, S., ... & Singhasivanon, P. (2010). Directly-observed therapy (DOT) for the radical 14-day primaquine treatment of Plasmodium vivax malaria on the Thai-Myanmar border. Malaria journal, 9(1), 1.
78. Chu, C. S., & White, N. J. (2016). Management of relapsing Plasmodium vivax malaria. Expert review of anti-infective therapy, 14(10), 885-900.
79. Looareesuwan S, White NJ, Chittamas S, et al. High rate of  Plasmodium vivax relapse following treatment of falciparum  malaria in Thailand. Lancet. 1987;2(8567):1052–1055.
80. WWARN Artemisinin based Combination Therapy (ACT) Africa Baseline Study Group, Clinical determinants of early parasitological response to ACTs in African patients with uncomplicated falciparum malaria: a literature review and meta-analysis of individual patient data. *BMC Medicine* 2015, 13:212 doi:10.1186/s12916-015-0445-x PMID: [26343145](http://www.ncbi.nlm.nih.gov/pubmed/26343145)
81. Bousema T, Okell L, Shekalaghe S, et al. Revisiting the circulation time of Plasmodium falciparum gametocytes: molecular detection methods to estimate the duration of gametocyte carriage and the effect of gametocytocidal drugs. Malaria Journal. 2010;9:136. doi:10.1186/1475-2875-9-136.
82. Mahande A, Mosha F, Mahande J, Kweka E. Feeding and resting behaviour of malaria vector, Anopheles arabiensis with reference to zooprophylaxis. Malaria Journal. 2007;6:100. doi:10.1186/1475-2875-6-100.
83. I. O. Oyewole, T. S. Awolola, C. A. Ibidapo, A. O. Oduola, O. O. Okwa, J. A. Obansa Behaviour and population dynamics of the major anopheline vectors in a malaria endemic area in southern Nigeria. J Vector Borne Dis. 2007 Mar; 44(1): 56–64.
84. Walker PGT, Gri n JT, Ferguson NM, Ghani AC. Estimating the most e cient allocation of interventions to achieve reductions in Plasmodium falciparum malaria burden and transmission in Africa: a modelling study. Lancet Glob Health 2016; published online June 3. http://dx.doi.org/10.1016/S2214-109X(16)30073-0.
85. Aguas, R., et al., *Prospects for malaria eradication in sub-Saharan Africa.* PLoS One, 2008. **3**(3): p. e1767.
86. Silal, S.P., et al., *Towards malaria elimination in Mpumalanga, South Africa: a population-level mathematical modelling approach.* Malaria Journal, 2014. **13**(1): p. 297-297.
87. Silal, S.P., et al., *Hitting a Moving Target: A Model for Malaria Elimination in the Presence of Population Movement.* PLoS One, 2015. **10**(12): p. e0144990.
88. Shretta, R., Silal, S.P., Malm, K*. et al. Estimating the risk of declining funding for malaria in Ghana: the case for continued investment in the malaria response.*Malaria Journal 19, 196 (2020). https://doi.org/10.1186/s12936-020-03267-9
89. S*ilal SP, Shretta R, Celhay OJ*et al.*Malaria elimination transmission and costing in the Asia-Pacific: a multi-species dynamic transmission model [version 2; peer review: 1 approved, 1 approved with reservations, 2 not approved]. Wellcome Open Res 2019, 4:62 (*[*https://doi.org/10.12688/wellcomeopenres.14771.2*](https://doi.org/10.12688/wellcomeopenres.14771.2)*)*
